# Power Transmission Tower Series Extraction in PolSAR Image Based on Time-Frequency Analysis and A-Contrario Theory

**DOI:** 10.3390/s16111862

**Published:** 2016-11-05

**Authors:** Dongqing Peng, Haijian Zhang, Wei Guo, Wen Yang

**Affiliations:** School of Electronic Information, Wuhan University, Wuhan 430072, China; pengdongqing@whu.edu.cn (D.P.); wei_guo_xd@163.com (W.G.); yangwen@whu.edu.cn (W.Y.)

**Keywords:** time-frequency analysis, a-contrario theory, polarimetric SAR, target extraction, cell-averaging constant false alarm rate

## Abstract

Based on Time-Frequency (TF) analysis and a-contrario theory, this paper presents a new approach for extraction of linear arranged power transmission tower series in Polarimetric Synthetic Aperture Radar (PolSAR) images. Firstly, the PolSAR multidimensional information is analyzed using a linear TF decomposition approach. The stationarity of each pixel is assessed by testing the maximum likelihood ratio statistics of the coherency matrix. Then, based on the maximum likelihood log-ratio image, a Cell-Averaging Constant False Alarm Rate (CA-CFAR) detector with Weibull clutter background and a post-processing operator is used to detect point-like targets in the image. Finally, a searching approach based on a-contrario theory is applied to extract the linear arranged targets from detected point-like targets. The experimental results on three sets of PolSAR data verify the effectiveness of this approach.

## 1. Introduction

Synthetic Aperture Radar (SAR) imaging is unrestricted by the weather conditions, which can make up for the blind spot caused by limits in time and space of the optical sensor. Hence, SAR images have special advantages in monitoring the physical conditions of Power Transmission Towers (PTT) under the condition of large scale natural disasters, such as earthquakes, floods, snow and so on [1,2,3]. PTT are mainly made of metal materials, thus generating a strong backscattering to electromagnetic waves, and this is helpful to find and locate the PTT in SAR images. When measuring the scattering characteristics of targets, the Polarimetric SAR (PolSAR) image contains more information than the single channel SAR image, which is more favorable for the target detection. As for the speckle noise, Polarimetric Whitening Filter (PWF) is not sufficient in most situations. Considering the non-stationarity of PTT, the Time-Frequency (TF) decomposition technique [4,5] can be used to analyze its scattering behavior in different angles and frequencies.

Thus far, there are few applications of SAR images in transmission corridor monitoring. In 1995, PWF was firstly carried out on the fully PolSAR image by Novak et al. [6]. Sarabandi et al. proposed a statistical polarization detection operator, conducting research on power line extraction using millimeter wave PolSAR image [7]. Liao et al. accurately identified the PTT target and the direction of PTT series from airborne SAR images of the flooded area [8]. A three-step approach of PTT extraction for PolSAR images with multi-resolution statistic energy level methods and a searching method based on a generic algorithm was presented in [9]. In [10], point-like targets were detected in the helix scattering component and then Radon transform was used to extract PTT. A different approach based on an adaptive region classification Constant False Alarm Rate (CFAR) detector was shown in [11,12] to detect point-like targets. The extraction results were then obtained through an energy minimization process applying Markov random field. In addition, there are some models adopted to PTT detection, such as the G0 statistical clustering model [13] and spherically invariant random vector model [14]. However, the results of general preprocessing methods (like multi-look processing and spatial filtering) when using CFAR to detect point targets are not satisfactory under complex ground scenes [15,16].

**Figure 1:**
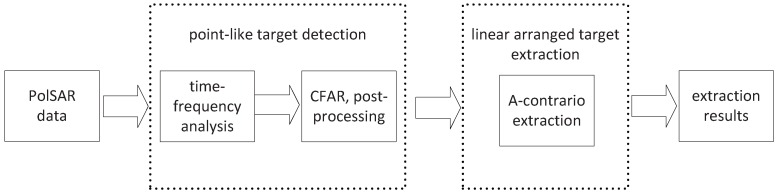
The block diagram of PTT target extraction from a PolSAR image.

Owing to the complexity of ground scene, there is great difficulty in directly detecting PTT series according to its scattering properties. It is necessary to combine shape and structure information. In this paper, the extraction of PTT series is divided into two steps: point-like targets detection; and linear arranged targets extraction. Point-like targets are detected by time-frequency analysis and a Cell-Averaging CFAR (CA-CFAR) detector [17]. Through time-frequency decomposition, the region with non-stationary scattering behavior can be detected as the polarimetric information is fully utilized. With the feature of linear arrangement, a-contrario theory, which was successfully applied in ship target detection from SAR images [18] is applied to extract PTT series. The flowchart of PolSAR PTT series extraction based on time-frequency analysis and a-contrario theory is shown in Figure 1.

The rest of this paper is organized as follows. Section 2 describes the process of point-like targets detection. Section 3 introduces the principle of a-contrario theory. Section 4 presents the experimental results. The conclusions are given in Section 5.

## 2. Point-Like Target Detection

### 2.1. 2D TF Analysis

In the process of SAR imaging, the full resolution image is formed by the superimposition of low resolution echoes obtained from different viewing angles. Thus, a pixel in an SAR image represents an integrated response in a certain observation angle range related to the orientation of the antenna. For high resolution data, the linear frequency modulation signal bandwidth of SAR system transmitting and receiving is relatively large. Thus, the received signal can be considered as multi-frequency, including the response characteristics of targets at multiple frequencies. TF analysis of the sub-band spectrum independently in range and azimuth directions not only can be used for the detection of anisotropic targets (including scatterers with complex geometry, artificial targets and linear arranged strong scatterers) and targets whose scattering characteristics are sensitive to frequency (such as cylinder targets, periodic structure or coupled scatterers), but can also be used to find non-stationary regions in distance-azimuth spectrums [19,20]. In this paper, the TF decomposition is based on two-dimensional (2D) short-term Fourier Transform [21].

#### 2.1.1. TF Decomposition

**Figure 2:**
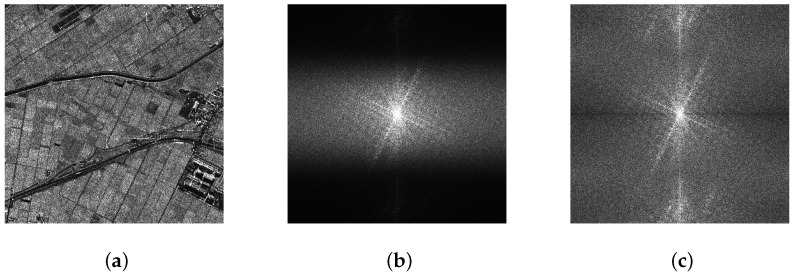
An example of the TF decomposition process. (**a**) the data of the HH channel; (**b**) the spectrum of the HH channel; and (**c**) the spectrum after compensation.

**Figure 3:**
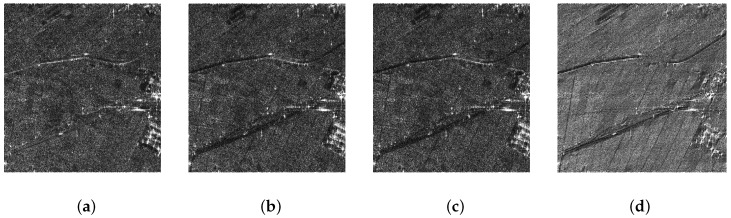
An example of TF decomposition result (*Q* = 4). (**a**) sub-image 1; (**b**) sub-image 2; (**c**) sub-image 3; and (**d**) sub-image 4.

By a convolution of 2D signal s(l) and analyzing function h(l), the transformation decomposes the 2D signal into different components, as follows [22]:

(1) $$\begin{array}{cc} s(l_{0};\omega_{0})= & \int s\left(l\right)h\left(l-l_{0}\right)e^{j\omega_{0}\left(l-l_{0}\right)}dl, \end{array}$$

where l=[x,y] is the coordinate of 2D spatial domain, $\omega_{0}=\left[\omega_{0az},\omega_{0rg}\right]$ represents the position in 2D frequency domain, and $s(l_{0};\omega_{0})$ indicates the decomposition result. By applying Fourier Transform to Equation (1), the spectrum of $s(l_{0};\omega_{0})$ is obtained:

(2) $$\begin{array}{cc} S(\omega ;\omega_{0})= & S\left(\omega \right)H\left(\omega ;\omega_{0}\right), \end{array}$$

which shows that the TF method can be used to describe the characteristics of signal spectral components selected by analyzing function h(l) in the spatial domain. The spatial domain and frequency domain are not independent, and their product meets the Heisenberg–Gabor uncertainty principle [22]:

(3) $$\begin{array}{cc} \Delta \omega \Delta l= & u, \end{array}$$

where *u* is a constant determined by h(l). Therefore, an appropriate analyzing function h(l) is needed to keep the spatial resolution that permits detail discrimination while being able to maintain a sufficiently low side lobe amplitude. The specific steps of decomposition are as follows:

- Applying 2D Fourier Transform to each polarimetric channel of PolSAR data (as shown in Figure 2a), the spectrum of each polarization channel is obtained, as shown in Figure 2b.
- Compensation of weighting functions applied during SAR focusing [19,20,21], as shown in Figure 2c.
- According to the set of sub-spectrum number Q, the spectrum is divided into Q sub-spectra.
- Multiplied with a weighting function, each sub-spectrum then is set back to the spatial domain using a 2D inverse Fourier Transform in order to get sub-images, as shown in Figure 3a–d.

#### 2.1.2. Second Order Statistics

In the case of single target backscattering, the reflection wave is connected with the incident wave through scattering matrix S. With elements determined by the characteristics of the target (especially the geometric and dielectric properties), the frequency of the wave, and the incident frequency, a scattering matrix S can fully describe the polarimetric response of a scatterer. In an orthogonal polarization basis, there is:

(4) $$\begin{array}{c} S=\left[\begin{array}{cc} S_{HH} & S_{HV}\\ S_{VH} & S_{VV} \end{array}\right]. \end{array}$$

For reciprocal medium, the scattering matrix has only three independent complex elements and can be transmitted into a scattering vector $k_{i}$ [21,23]:

(5) $$\begin{array}{c} k_{i}=\frac{1}{\sqrt{2}}{\left[\begin{array}{ccc} S_{HH}+S_{VV} & S_{HH}-S_{VV} & 2S_{HV} \end{array}\right]}^{T},\;i=1,...,Q, \end{array}$$

where *T* stands for transpose operator.

After TF decomposition, the polarimetric information of each sub-image is gathered by a polarimetric TF vector [21,23]:

(6) $$\begin{array}{c} k_{TF}={\left[\begin{array}{cccc} k_{1}^{T}, & k_{2}^{T}, & \ldots , & k_{Q}^{T} \end{array}\right]}^{T}. \end{array}$$

Then, a polarimetric TF coherency matrix, $T_{TF}$, is computed as follows [21,23]:

(7) $$\begin{array}{c} T_{TF}=\langle k_{TF}k_{TF}^{*}\rangle =\left[\begin{array}{ccc} T_{11} & \cdots & T_{1Q}\\ \vdots & \ddots & \vdots \\ T_{Q1} & \cdots & T_{QQ} \end{array}\right], \end{array}$$

where superscript ${}^{*}$ indicates the transpose conjugate operator.

#### 2.1.3. Non-Stationary Media Detection and Analysis

Each pixel of the SAR image corresponds to a set of different coherent matrix samples extracted from the sub-band spectrum of different distances and azimuths. The stationary scattering characteristics of each pixel can be obtained by testing the statistics of the coherent matrix [24].

For Single Look Complex (SLC) data, the *n*-look 3×3 coherency matrix $T_{ii}$ (i=1,...,Q) follows a Wishart distribution $W_{C}\left(n,\Sigma_{ii}\right)$ (for SLC data, n=1). If the coherent matrix of Q sub-band spectrum of a pixel has the same distribution and satisfies the following assumptions:

(8) $$\begin{array}{c} \Sigma_{11}=\Sigma_{22}=\cdots =\Sigma_{QQ}, \end{array}$$

then the pixel is considered to have the characteristic of an isotropic spectrum. By using the maximum likelihood ratio Λ of each independent coherent matrix, the above assumptions are tested:

(9) $$\begin{array}{c} \begin{array}{cc} \Lambda & =\frac{\prod_{i=1}^{Q}{\left|T_{ii}\right|}^{n_{i}}}{{\left|T_{3t}\right|}^{n_{t}}},\\ \;\mathrm{with}\;n_{t} & =\sum_{i=1}^{Q}n_{i},T_{3t}=\frac{1}{n_{t}}\sum_{i=1}^{Q}n_{i}T_{ii}, \end{array} \end{array}$$

where $n_{i}$ represents the number of scattering vectors $k_{i}$ used to calculate $T_{ii}$.

**Figure 4:**
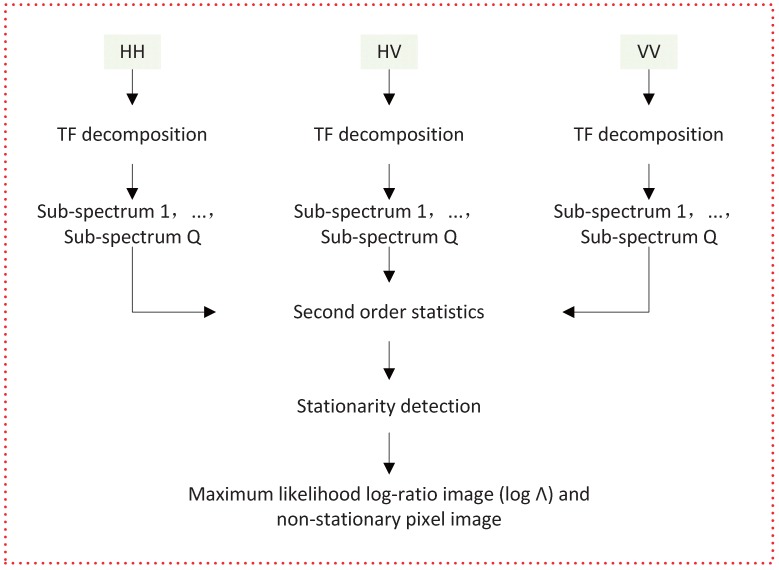
The process of 2D TF analysis.

A false alarm probability $P_{fa}\left(c_{\beta }\right)$ is chosen arbitrarily. The assumption is right if $\Lambda >c_{\beta }$, with $P_{fa}\left(c_{\beta }\right)=P\left(\Lambda \leq c_{\beta }\right)=\beta$, and the target is considered to be isotropic; otherwise, the target is anisotropic. So far a non-stationary pixel image is obtained. Figure 4 describes the process of 2D TF analysis, which is equivalent to doing preprocessing on SAR image before the detection of point-like targets.

### 2.2. CA-CFAR Point-Like Target Detector

A CFAR detector needs to know the Probability Density Function (PDF) of clutter and threshold, which depends on the constant false alarm rate $P_{fa}$. Generally, Rayleigh PDF and Gamma PDF are widely used in the clutter model. However, for the ground clutter with a long tail, a better model is demanded [25]. Here, a Weibull PDF is applied, with the density function as follows:

(10) $$\begin{array}{c} f\left(x\right)=\frac{c}{b}{\left(\frac{x}{b}\right)}^{2}e^{-{\left(\frac{x}{b}\right)}^{c}}, \end{array}$$

where *x* is the data amplitude, *b* and *c*, respectively, represent the scale and shape parameter which can be estimated from the data mean <x> and standard deviation $\sigma_{x}$, $c=0.0791{\left(\frac{<x>}{\sigma_{x}}\right)}^{2}+0.8481\left(\frac{<x>}{\sigma_{x}}\right)+0.0817$ and $b=\frac{<x>}{\Gamma (1+\frac{1}{c})}$ [26]. For a given $P_{fa}$, we can get the detection threshold:

(11) $$\begin{array}{c} T=b\cdot \sqrt[{c}]{In(P_{fa})}. \end{array}$$

To calculate the detection threshold adaptively, a sliding window is designed. A CA-CFAR detector is applied to detect point-like targets and generate a binary image, in which value 1 represents point-like targets while value 0 represents clutter pixel. Since isolated pixels are less likely to be true targets, a morphological open operator [27], aiming at removing isolated pixels, is applied to reduce point-like targets, which simplifies the further extraction. Finally, a post-processing image is produced.

## 3. Linear Arranged Target Extraction Based on A-Contrario Theory

In the post-processing image, assuming the number of pixels with value 1 is *N*, the point-like targets are contained in domain *D*. Considering the linear arranged feature of PTT series, we focus on the extraction of point groups which are linear arranged in the domain *D*. Hence, an extraction method based on a-contrario theory is illustrated in the section [28].

### 3.1. A-Contrario Hypothesis

Human eyes can identify linear arranged targets intuitively since the human visual perception system groups pixels together to form structures that people see according to geometric criteria. A-contrario theory acts exactly as the criteria when a computer detects the linear arranged targets. Its decision framework is the visual grouping principle based on Helmholtz, in which events that have a large deviation from the stochastic model are visually meaningful [29].

Helmholtz principle: if the probability that a structure (such as a line or curve) is randomly arranged by points is very small, the structure can be easily found. That is to say, if the occurrence probability of a structure is very small, then the structure is not randomly generated, and it is of great significance.

A-contrario hypothesis $H_{0}$ is put forward: as the result of random processes, point-like targets with a value of 1 are independent and uniformly distributed in domain *D*. In the case that background points do not exactly follow the hypothesis $H_{0}$, a-contrario theory still works as point groups with low occurrence probability can be detected by computer. Assume a geometric structure *E* corresponded to one of the point groups, with the set of point groups defined as $E_{s}$.

Then, the number of false alarms is defined as the probability that the geometric structure *E* appears under $H_{0}$. Under the hypothesis $H_{0}$, the probability of at least *k* points among the *N* points falling into the geometric structure *E* is organized as follows:

(12) B(N,k,p)=∑i=kNNipi(1−p)N−i,

where *p* is the probability that one point falls into *E*.

The number of false alarms of *E* is defined as follows:

(13) $$\begin{array}{c} NFA\left(E\right)=N_{total}\cdot B\left(N,k,p\right), \end{array}$$

where $N_{total}$ is the possible number of geometric structures *E* formed by all point groups. When the value of NFA(E) is very large, it indicates that the linear arranged point group in geometric structure *E* is likely to be an accidental arrangement. Thus, it is less likely to be a true target. Similarly, it is probably the true target when the value of NFA(E) is very small. A threshold of NFA(E) is set as *ε*, and if NFA(E)≤ε, the corresponding point group in *E* will be extracted as linear arranged targets [30].

### 3.2. A Reasonable Geometric Structure

The occurrence probability of geometric structure *E* under hypothesis $H_{0}$ determines whether the point groups inside *E* are extracted as linear arranged targets or not. Hence, the extraction accuracy is relevant to geometric structure *E* [28]. Strip method is considered to extract the linear arranged point groups in PolSAR images. Targets that need to be extracted are some points in PolSAR images, and then the linear arranged point group can be defined as many points in a rectangle. The more slender the rectangle, the more accurate the extraction.

**Figure 5:**
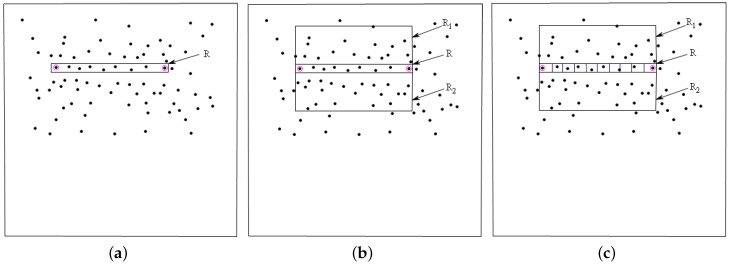
(**a**) a schematic representation of rectangle *R*; (**b**) a local rectangle window covered *R* with three parts; and (**c**) the rectangle *R* divided into *C* parts.

As shown in Figure 5a, the geometric structure *E* is defined as a group of points covered with a thin rectangle *R*. The width of *R* is the distance between the two points marked by red circles. Given a max ratio of width and height, different heights smaller than the max height (with width fixed) are tested. However, if all points in domain *D* are distributed on the same side, it can be conferred that most of the point groups inside their corresponding geometric structure *E* have low occurrence probability under hypothesis $H_{0}$. Thus, the geometric structure *E* is developed as shown in Figure 5b, where the rectangle *R* is covered by a local rectangle window with the same width as *R*. Additionally, the local window consists of three parts: *R*, $R_{1}$ and $R_{2}$. *R* is the original rectangle; $R_{1}$ and $R_{2}$ are of the same size. Given a max ratio of width and height, different widths will be tested. To avoid the side effect, the total number of points inside *R*, $R_{1}$ and $R_{2}$ are defined as [28]:

(14) $$\begin{array}{c} n\left(R,R_{1},R_{2}\right)=2max\left(M_{1},M_{2}\right)+M, \end{array}$$

where *M*, $M_{1}$ and $M_{2}$ are the total number of points inside *R*, $R_{1}$ and $R_{2}$, respectively, and max represents maximum function.

To avoid the effect of point clusters inside *E*, the rectangle *R* is divided into *C* small grids $B_{i}$ (i=1,...,C) with the same size, as shown in Figure 5c. $C=\sqrt{N}$ different values are tested to obtain the lowest NFA(E). With *L* local windows, the total number of tests is [28]:

(15) $$\begin{array}{c} N_{test}=\frac{N(N-1)}{2}WLC=\frac{N(N-1)}{2}WL\sqrt{N}, \end{array}$$

where *W* is the number of widths and *L* is the number of local windows.

Assume the area of rectangle *R* is $S_{R}$, and the area of grid $B_{i}$ is $S_{B_{i}}$. The probability of a point in window *R* falling into grid *B* is $p_{0}=\frac{S_{B_{i}}}{S_{R}}$. For any grid $B_{i}$, the probability of at least one of the $n(R,R_{1},R_{2})$ points included in the grid is [28]:

(16) $$\begin{array}{c} p_{1}\left(R,C\right)=1-{\left(1-p_{0}\right)}^{n(R,R_{1},R_{2})}. \end{array}$$

Grids including at least one point are called occupied grids, with the number of occupied grids denoted as b(R,C), the probability that at least b(R,C) grids of the *C* grids are occupied is $B(C,b\left(R,C\right),\;p_{1}\left(R,C\right))$ and the number of false alarms of *E* organized as follows:

(17) $$\begin{array}{c} NFA\left(E\right)=\frac{N(N-1)}{2}WL\sqrt{N}\cdot B\left(C,b\left(R,C\right),p_{1}\left(R,C\right)\right). \end{array}$$

## 4. Experimental Results and Analysis

**Figure 6:**
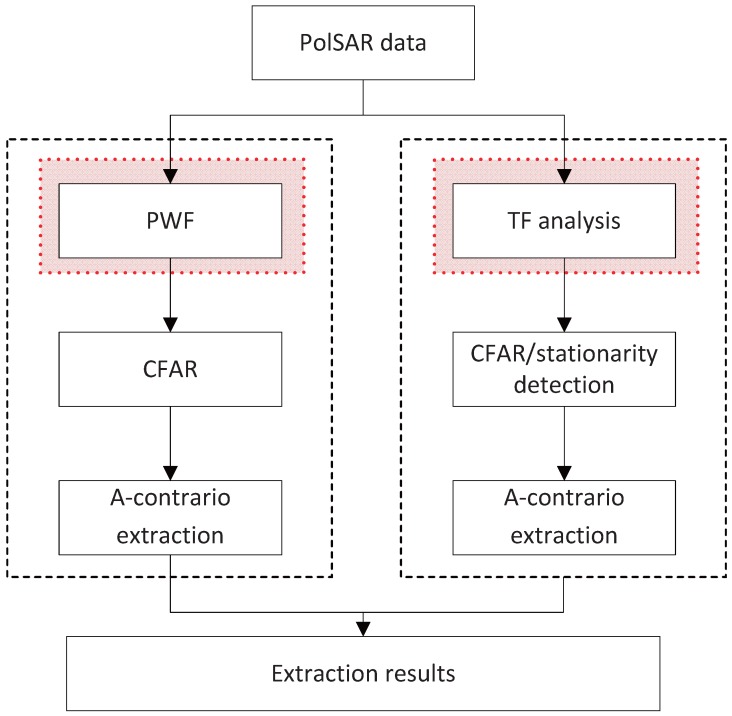
The flowchart of PTT series extraction.

To verify the effectiveness of the proposed approach, three groups of experiments are performed in this section. The three sets of data are Niigata Site Area of Japan, Inner Mongolia Area of China and Linshui City in Hainan Province of China data, respectively. When detecting point targets, PWF is used for a comparison. The specific flowchart of a PWF based approach and TF based approach is shown in Figure 6.

### 4.1. Experiment I

**Figure 7:**
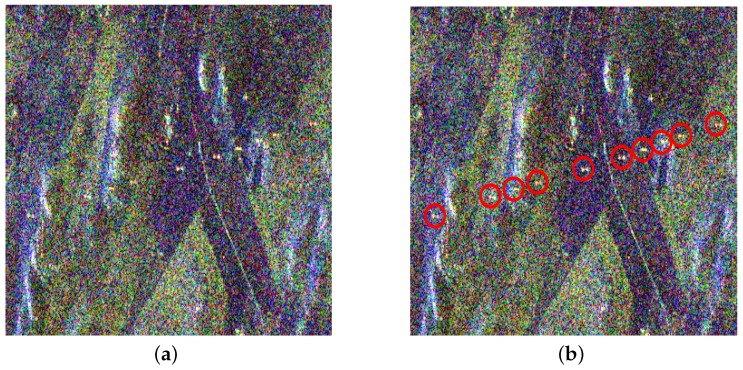
Pauli color coded image of Inner Mongolia. (**a**) original PolSAR image; and (**b**) marked image.

C-land PolSAR images acquired by RADARSAT-2 with 56 mm wavelength and 5.4 GHz frequency over Inner Mongolia, China, as shown in Figure 7a, are used in this experiment. The original data has 6296 × 2492 pixels and the spatial resolution is 8 m × 8 m. We select a sub-image with 400 × 400 pixels to perform the experiment, in which PTT targets are marked by red circles, as shown in Figure 7b.

The sub-spectrum number *Q* of TF decomposition is set as four, and then four sub-images are obtained after TF decomposition. To test the stationarity of each pixel, the maximum likelihood ratio is calculated according to the coherency matrix of sub-images. Then, point-like targets are detected through a CFAR detector. If there are too many candidate targets, not only the extraction speed but also the extraction results will be affected. Thus, a post-processing operator is applied to further reduce the candidate targets. Finally, linear arranged PTT targets are extracted using a-contrario method. The detailed parameter setting is shown in Table 1.

**Figure 8:**
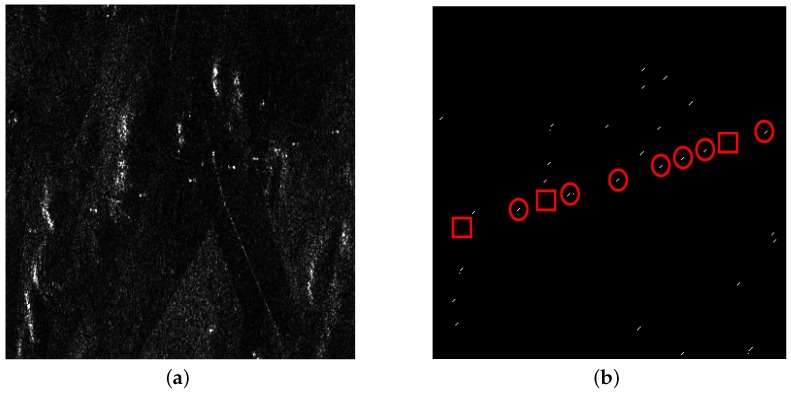

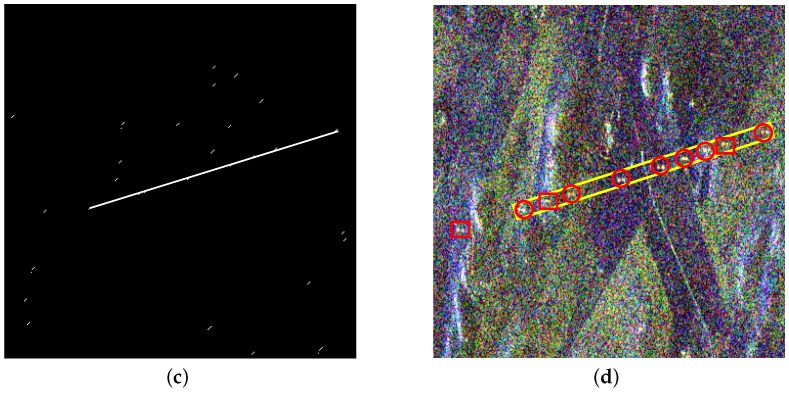
(**a**) filtered image based on PWF; (**b**) point-like target detection results; (**c**) a-contrario extraction result; and (**d**) extraction result marked on the original image.

**Figure 9:**
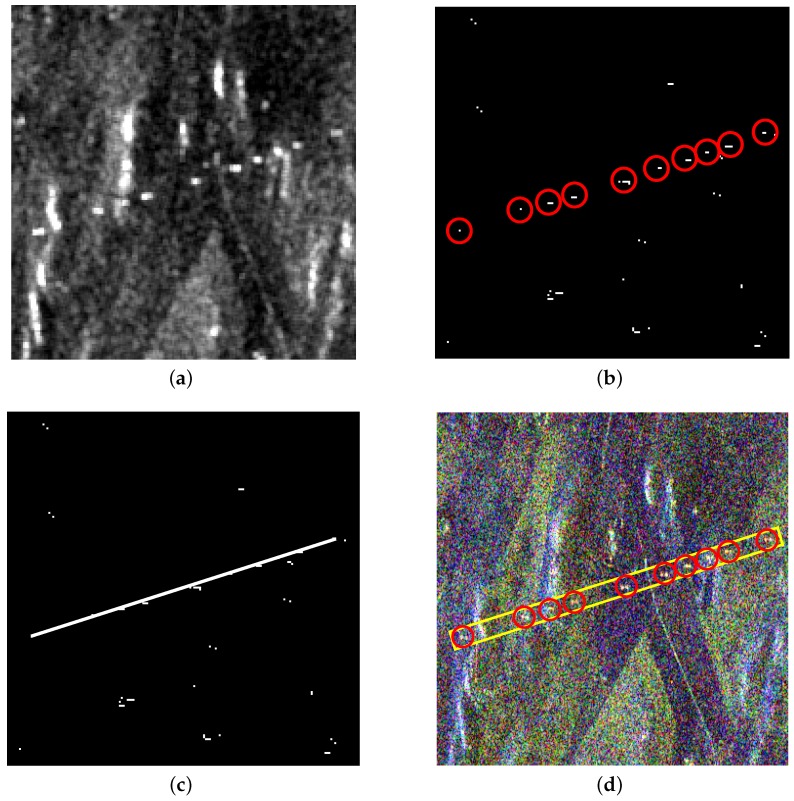
(**a**) filtered image based on TF analysis; (**b**) point-like target detection results; (**c**) a-contrario extraction result; and (**d**) extraction result marked on the original image.

From Figure 8a and Figure 9a, it can be seen that there are also some highlighted areas besides PTT targets, which are mostly ridges. Here, candidate targets are made up of PTT targets, ridges and other highlighted areas. Affected by noise and ground scenes, detection after PWF loses three PTT targets, as shown in Figure 8b, marked by red rectangles. Replacing the PWF with TF analysis, all PTT targets are detected, as shown in Figure 9b. Table 2 shows that the TF analysis involved method has a higher detection rate of PTT targets than the PWF involved method. Through a-contrario extraction, linear arranged PTT targets are successfully extracted by both methods, as shown in Figure 8c and Figure 9c. However, linear arranged PTT targets extracted by method one are incomplete owing to a loss of PTT targets in point-like targets detection, while method two shows a better result, as shown in Figure 8d and Figure 9d. Detected PTT targets, lost PTT targets and *R* in geometric structure *E* are marked by red circles, red rectangles and yellow rectangles, respectively.

### 4.2. Experiment II

**Figure 10:**
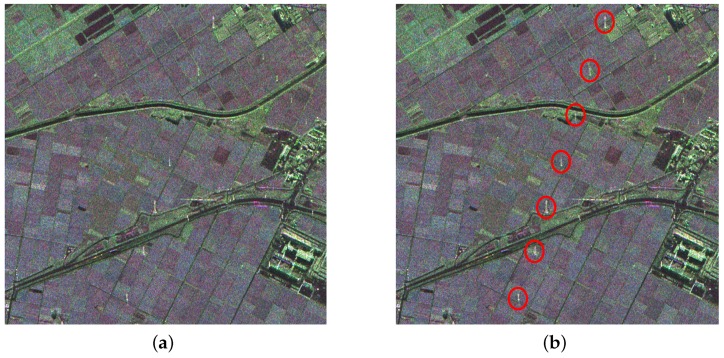
Pauli color coded image of Niigata Site Area. (**a**) original PolSAR image; and (**b**) marked image.

The data of experiment II is a part of L-band Pi-SAR images in the Niigata Site Area, as shown in Figure 10a. The original image size is 4000×4000, and the spatial resolution is 3 m × 3 m. With a lot of ground objects included, roads, urban construction and other non-natural targets will bring great difficulty to the detection. An area with 1000×1000 pixels is cut out for the experiment, containing PTT targets marked by red circles, as shown in Figure 10b.

Considering the complexity of ground scene in this data, a CA-CFAR detector is applied to the maximum likelihood log-ratio image derived from TF analysis (Q=4), and then the detection result is used for the same further processing as in experiment I. Related parameter settings are shown in Table 3.

**Figure 11:**
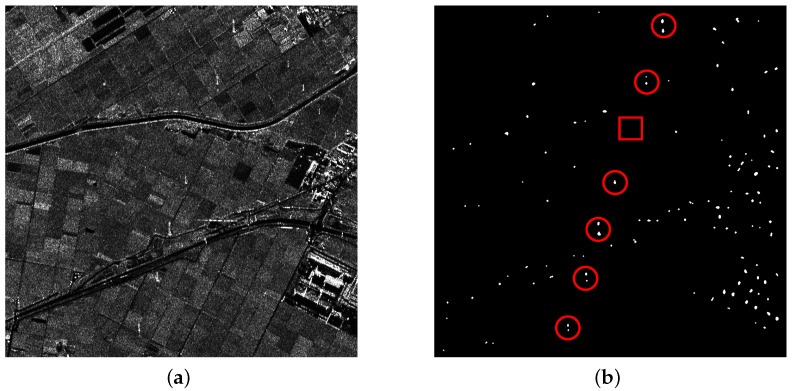
(**a**) filtered image based on PWF; and (**b**) point-like target detection results.

**Figure 12:**
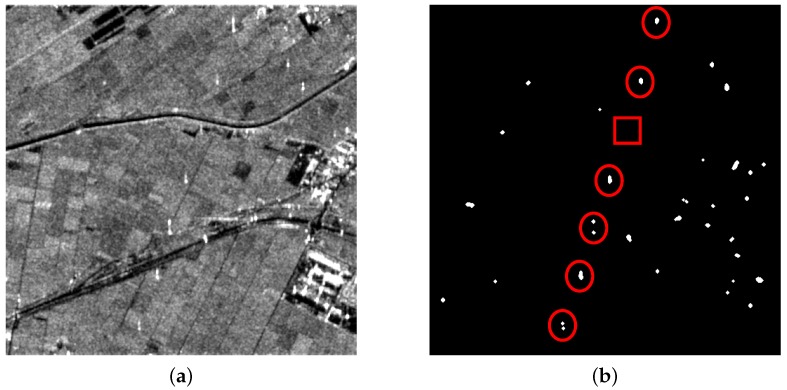

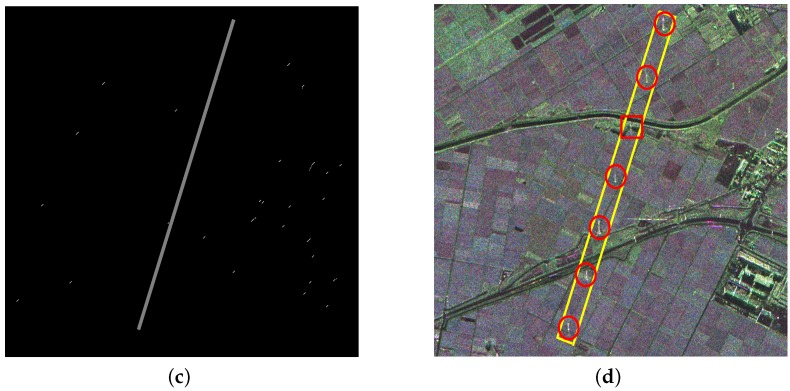
(**a**) filtered image based on TF analysis; (**b**) point-like target detection results; (**c**) a-contrario extraction result; and (**d**) extraction results marked on the original image.

With many non-natural scenes (like roads and buildings) in the data, there are a lot of candidate targets that resulted from point-like target detection. Targets marked by red circles and rectangles in Figure 10b are PTT targets and weak PTT targets, respectively. In a complicated background, the weak target is missing in both methods, as shown in Figure 11b and Figure 12b marked by red rectangles. Along the road, there exist many candidate targets. The greater the number of candidate targets, the greater the interference on extraction. Hence, candidate targets should be as few as possible on the premise that all PTT targets are detected. Stationarity detection based on TF analysis can effectively remove some candidate targets because PTT targets are non-stationary while some candidate targets are stationary. Since candidate targets in Figure 12b are much less than in Figure 11b, it can be inferred that the effect of PWF is limited for complex ground scenes. In Table 4, two methods show the same detection rate of PTT targets. However, the PWF involved method failed to extract the linear arranged PTT targets due to the great interference coming from a high false alarm rate of CFAR. The false alarm probability of point-like targets is a constant. In a ground scene, there are many kinds of point-like targets, including PTT. The high false alarm rate of CFAR resulted from other point-like targets. Linear arranged PTT targets are successfully extracted by a TF involved method despite losing one weak PTT target, as shown in Figure 12c,d. The experimental results verify the feasibility and effectiveness of the detection approach based on TF analysis and a-contrario theory.

### 4.3. Experiment III

**Figure 13:**
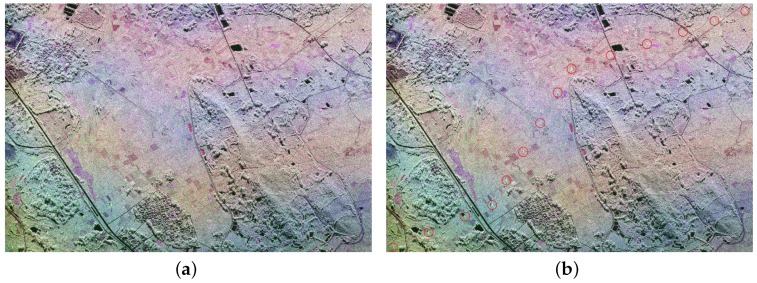
Pauli color coded image of Linshui City in Hainan Province. (**a**) original PolSAR image; and (**b**) marked image.

PolSAR data used in this section is acquired in X-band by the 38th Research Institute of China Electronics Technology Group Corporation (CETC38) single-baseline PolInSAR system over Linshui City in Hainan Province of China, with a spatial resolution of approximately 0.5 m × 0.5 m and 3380×4990 pixels, as shown in Figure 13a. PTT targets in the image are piecewise linear arranged, as shown in Figure 13b, marked by red circle.

After pre-processing (TF with Q=4 and PWF), CA-CFAR is applied to detect point-like targets. Considering that PTT targets in the image are piecewise linear arranged, segment detection based on a-contrario theory is carried out, roughly divided into three sections. Related parameters are shown in Table 5.

**Figure 14:**
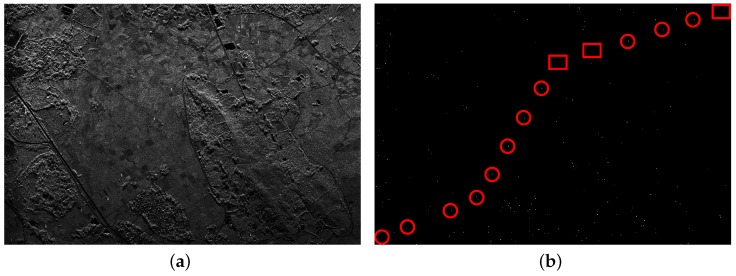
(**a**) filtered image based on PWF ; and (**b**) point-like target detection results.

**Figure 15:**
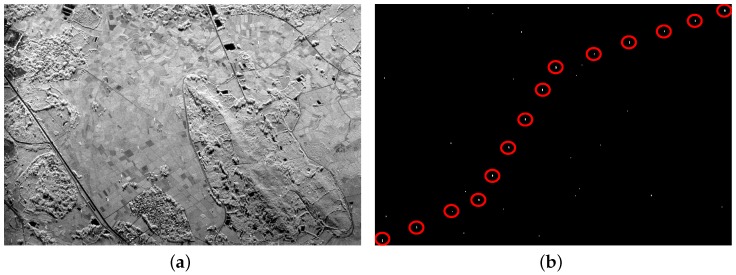

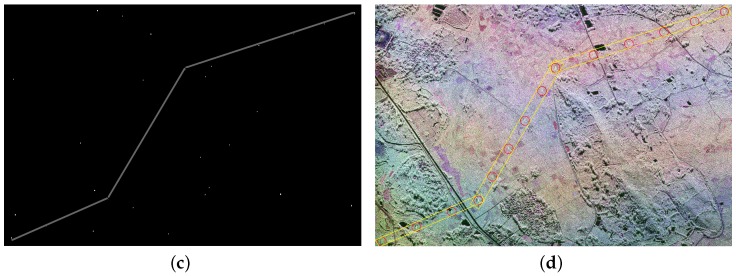
(**a**) filtered image based on TF analysis (**b**) point-like target detection results; (**c**) a-contrario extraction results; and (**d**) extraction results marked on the original image.

In this image, there exist 14 PTT targets. Under the condition that CA-CFAR parameters are the same, PWF based CA-CFAR detection loses three PTT targets, with many false alarm point-like targets, as shown in Figure 14a,b, while TF analysis based CA-CFAR detection get all PTT targets detected, with much less false alarm point-like targets, as shown in Figure 15a,b. With a high false alarm rate (targets wrongly detected as PTT), as shown in Table 6, further measures are necessary, thus using a-contrario extraction, which is state-of-the-art in point alignment detection [28]. The PWF based method failed to extract the PTT series, resulting from a high false alarm rate (over 90%). Through TF analysis, the false alarm rate is relatively low. The TF based method finished the extraction, as shown in Figure 15c,d.

In three experiments, a CA-CFAR detector can only detect point-like targets rather than distinguish PTT targets from other point-like targets. This is the reason why a-contrario extraction is applied. A parameter (false alarm rate), closely related to the first step (PWF or TF), has a great influence on a-contrario extraction. From experimental results, TF analysis is superior to PWF in reducing false alarm rate, and it can be inferred that a-contrario extraction is effective when the false alarm rate is below a value that needs to be estimated from numerous experiments.

With many false alarm targets, other lines in addition to the PTT line may be extracted. For this problem, the adoption of TF analysis got a relatively low false alarm rate, reducing the probability of lines formed by false alarm targets. In addition, isometric features can be considered in a-contrario extraction, avoiding this situation to a large extent. In our three experiments, this phenomenon does not appear.

Table 7 records the time cost of two methods in experiment III. The computation time is recorded by implementing the Matlab code on a PC with a 3.20 GHz Intel Core i5 Processor. In the first step, TF analysis takes more time than PWF, but the TF decomposition reduced the number of pixels to be processed, decreasing the computation cost of CA-CFAR, which is time-consuming. Hence, TF based CA-CFAR is much faster than PWF based CA-CFAR.

## 5. Conclusions

Based on TF analysis and a-contrario theory, this paper presents a new approach for PTT series extraction in PolSAR Images. Taking advantage of the non-stationary scattering characteristics of PTT targets, TF analysis is applied to remove some stationary candidate targets while point-like targets with PTT targets included are detected, thus decreasing the interference for the subsequent a-contrario extraction. Despite losing individual PTT targets with weak scattering characteristics, experiments on three sets of PolSAR data successfully extract the linear arranged PTT targets. From the time cost, our method greatly reduced the computation cost by the use of TF analysis, thus saving the time cost and confirming the feasibility of our method. For urban areas, the existence of many point-like targets may cause performance degradation of the method. In our experiments, including local urban area, this method works well.

## Figures and Tables

**Table 1:** Parameter setting of Experiment I.

False alarm probability	0.001	Min number of points in a rectangle	5
The size of target window	1×1	Max number of false alarm	1
The size of protect window	2×2	Min rectangle width	1
The size of clutter window	4×4	Max ratio of width and height	20

**Table 2:** Results comparison of two methods in Experiment I.

Method	Total Number of PTT Targets	The Number of Detected PTT Targets	PTT Target Detection Rate	False Alarm Rate of CFAR (Targets Wrongly Detected as PTT)	Whether PTT Series Are Extracted Successfully
PWF + a-contrario	10	7	70%	77.42%	Yes
TF + a-contrario	10	10	100%	69.70%	Yes

**Table 3:** Parameter setting of Experiment II.

False alarm probability	0.001	Min number of points in a rectangle	5
The size of target window	2×2	Max number of false alarm	1
The size of protect window	4×4	Min rectangle width	1
The size of clutter window	8×8	Max ratio of width and height	20

**Table 4:** Results comparison of two methods in Experiment II.

Method	Total Number of PTT Targets	The Number of Detected PTT Targets	PTT Target Detection Rate	False Alarm Rate of CFAR (Targets Wrongly Detected as PTT)	Whether PTT Series Are Extracted Successfully
PWF + a-contrario	7	6	85.71%	>90%	No
TF + a-contrario	7	6	85.71%	75.76%	Yes

**Table 5:** Parameter settings of Experiment III.

False alarm probability	0.001	Min number of points in a rectangle	4
The size of target window	2×2	Max number of false alarm	1
The size of protect window	4×4	Min rectangle width	1
The size of clutter window	8×8	Max ratio of width and height	25

**Table 6:** Results comparison of two methods in Experiment III.

Method	Total Number of PTT Targets	The Number of Detected PTT Targets	PTT Target Detection Rate	False Alarm Rate of CFAR (Targets Wrongly Detected as PTT)	Whether PTT Series Are Extracted Successfully
PWF + a-contrario	14	11	78.57%	>90%	No
TF + a-contrario	14	14	100%	58.82%	Yes

**Table 7:** Time cost of two methods in Experiment III.

Method/Time Cost	First Step (PWF or TF)	Second Step (CA-CFAR)	Third Step (A-Contrario)	Total Time (The Sum of Three Steps)
PWF + a-contrario	23.78 s	76,345.38 s	–	76,369.16 s
TF + a-contrario	428.75 s	5299.54 s	1.74 s	5730.03 s

–: it indicates that the step failed.

## Abbreviations

The following abbreviations are used in this manuscript:

SAR	Synthetic aperture radar
PTT	Power transmission tower
PolSAR	Polarimetric synthetic aperture radar
PWF	Polarimetric whitening filter
TF	Time-frequency
CFAR	Constant false alarm rate
CA-CFAR	Cell-averaging constant false alarm rate
2D	Two-dimensional
SLC	Single-look complex
PDF	Probability density function
